# When worlds collide

**DOI:** 10.1186/1745-6215-10-108

**Published:** 2009-11-27

**Authors:** Clive E Adams, Mauricio Silva de Lima

**Affiliations:** 1 The Sir Colin Campbell Building, University of Nottingham Innovation Park, Triumph Road Nottingham, NG7 2TU, UK; 2 Eli Lilly UK and Ireland, Lilly House, Priestley Road, Basingstoke, Hampshire, RG24 9NL, UK

## Abstract

The UK has a strong tradition of innovative evaluative health care research. There are, however, considerable forces impeding collaboration between clinicians, academics, patients and their advocates and industry. This paper argues that, if the UK is to regain a position at the forefront of clinical research into evaluation of care, some of these forces need to be overcome. Now, with explicit encouragement from funders within the UK's NHS, it is urgent that all parties discover better ways of working together so that more broad and meaningful research can be produced in a timely fashion.

## Introduction

The National Health Service and the Welfare State have come to be used as interchangeable terms, and in the mouths of some people as terms of reproach. Why this is so it is not difficult to understand, if you view everything from the angle of a strictly individualistic competitive society. A free health service is pure Socialism and as such it is opposed to the hedonism of capitalist society. - Aneurin Bevan, In Place of Fear[1].

I have never known much good done by those who affected to trade for the public good. It is an affectation, indeed, not very common among merchants, and very few words need be employed in dissuading them from it. - Adam Smith, Glasgow Edition of Smith's works[2].

The authors are friends and colleagues who have worked together for years. Although career paths have been different we do not feel that these should be divergent. For that not to be the case, however, ways of working of all those concerned do need to change and mature. The timing for this is right with recent NHS initiatives. There are now explicit directives for relevant research within the NHS to be undertaken in close collaboration with industry, academia and consumers[3]. UK Clinical Research Collaboration (UKCRC), for example, is a partnership of organizations, including industry and consumers, focused on production of high-quality clinical research within the NHS. Although there are a number of shining examples of the success of collaboration, it is, in many circumstances, a problematic evolution fraught with misunderstandings and frustrations[4]. Clinicians may see research as often an academic or industrial exercise with little implication for practice in the real world. For some areas of health care this is, in no small measure, true. Industry may see the NHS as an inefficient leviathan[5] with many committed individuals but, as a whole, difficult to influence or bring to industry standard. Academia may understand some of the frustrations of research within the NHS but be, understandably, wary of becoming tainted with the reputation that goes along with accepting industry support. After all, in many instances, industry funding predicts results and, in any case, there is a well-recognized risk of debauchery associated with academics who work closely with industry[6,7]. In the face of the unresolved issues it has been easy for academics to retreat into ivory towers untainted by industry and untroubled by the needs of services. Finally consumers can find it difficult to have as strong and united a voice as that of health care professionals, industry or academics and can end up feeling disempowered and patronised.

These seemly different perspectives can lead to an uncomfortable low level of productivity in which sleaze, bias and inefficiency are common. Everyone loses. Industry becomes frustrated with the dearth of information forthcoming from the NHS' important [and wealthy] population and is more than tempted to go elsewhere to generate similar data. Clinicians become passive and cynical recipients of evidence generated from outside of the NHS. Academics are disempowered by living in ivory towers so far from the real world. Patients and their carers miss the opportunity to either become involved in studies or gain benefits from truly relevant and local research, and the NHS continues to struggle with avoidable inefficiencies.

## Discussion

There are great gains of working together. It should be feasible that the NHS remembers and respects the original altruism upon which it was built and encourages the opportunity of helping science move forward for the good of those served by the NHS. It would seem feasible that this also fits with the needs of industry, academia and consumers. All stakeholders, however, may need to change and to learn from the successes and failures of others. The NHS is increasingly supporting research time of its clinicians. This is still in the early stages but there is active and practical encouragement (funding) of clinicians having protected research time[8]. There is concrete recognition that research is part of an NHS job, is something to add variety to the clinical role, and is, potentially, a healthy addition for the workforce[9]. The NHS has also encouraged much clinically relevant research in swathes of grant giving across all health care specialties. Department of Health support is now even more explicitly for the direct benefit of the National Health Service[10] but also the genuine involvement [and not just consultation] of people who use the service. The NHS is again in the vanguard of encouraging real world, clinically meaningful research.

Academia may still be some distance behind. Too much work is still bid for or undertaken that can have little direct or even indirect relevance to people within the NHS[11]. There are still instances of an enormous cultural barrier where it has not been understood that some of the fine-grain issues that are of genuine interest to academia may not be so valuable to providers of care. Although, of course, there must be a balance struck between innovative, imaginative research and the practical needs of the NHS, there may still be many academics too far removed from practical patient care. Nevertheless, there are signs that academia, too, has responded to the NHS' encouragement to climb down from the tower - the NHS' lead [and money] has helped.

Industry, up to this point, has mostly worked with different priorities[5]. Developing an innovative medicine, preparation or device is a long-term and expensive enterprise. Most new medicines, for example, will fail on early phase clinical studies, mainly because of safety reasons. Others will not demonstrate clear cost benefits over existing treatments. When an innovative medicine reaches the regulatory approval process, specific study features and outcomes are demanded. Regulatory trials do not necessarily hold a robust cost-effectiveness model from the perspective of NICE or the general public. The final and most important customers in this complex chain are often ignored in industry trials - patients and their carers. Although industry has been knocking on the door of the NHS for years, in many cases this resulted in a relationship where the industry model of research has been imposed on relatively few receptive researchers. As a result, the examples of success are far fewer than would be expected. It does help when the interests of all stakeholders concur. Survival rates in people with cancer are of interest to everyone. In other sub-specialties, where the outcomes are not of equal interest to everyone, such as in mental health, the results of co-working are much fewer, less successful and less meaningful. When industry respects the needs of the NHS, the NHS' capacity as one of the largest single organisations providing health care, and the sheer size of the NHS 'business' [eclipsing most companies], then co-working has been proved to be fruitful[12]. Where these elements are not considered, both individuals and networks of individuals slip into an all too familiar uneasy and often sleazy relationship betraying principles upon the NHS was built.

Industry, academia, and even the NHS should learn from patients and practitioners about clinically relevant patient outcomes, and deliver solutions for their main unmet health needs. The UK government's call for a collaborative work between these providers might initially seem simplistic, but, rather, is a unique opportunity to the NHS, academia and industry to finally work for the people who ultimately should to be beneficiaries of collaborative research. The NHS has a fine tradition of imaginative clinical research that, now, by some measures, has fallen far behind some other countries in Europe. The Centre for Medicines Research data suggests that the UK market share of global patient recruitment fell from 6% in 2000 to around 2% in 2006 [13]. The same source also examined the number of patients in commercial trials and has found that the UK has declined from a third position in 2000 to ninth place in 2006. In terms of active sites, the Average Relative Annual Growth Rate of countries of commercial clinical trials showed the UK shrunk by 10% between 2000 and 2006. In the same period, other European countries like Spain (14.9%) and Germany (11.7%) achieved growth [14].

## Conclusion

Recent initiatives within the NHS, imposing a cultural change in academia and encouraging it in industry, make this time an exciting opportunity for new research initiatives. Of course cultural change takes time, and individuals and networks will continue to produce yet more studies of the type that have been seen for sixty years. In some subspecialties we are barely at the stage of talks about talks - but right across many health care specialties, talks there are, both formal and informal[15]. Perhaps no one will get all they want. Nevertheless, this initiative has a chance of pushing the research culture in the UK towards world-leading and ground breaking work again. The initiatives are opening the doors to the NHS to provide a further service to patients and their carers via their involvement in research, for academics to truly do imaginative and important work of clear relevance at the coal-face of care, as well as for industry to clean up a tarnished image and help gain practice-changing, evidence about the interventions they have invested so much in.

## List of abbreviations

NHS: National Health Service; NICE: National Institute for Clinical Excellence.

## Competing interests

Mauricio Silva de Lima own shares in Eli Lilly and Company.

## Authors' contributions

Both CA and MSDL conceived, carried out the literature search, and worked to draft the manuscript.

## References

[B1] Bevan Aplace of fear 1952 London: Heinemann

[B2] Smith A An enquiry into the nature and causes of the wealth of nations 1976 Oxford: Oxford University Press

[B3] Department of Health Best research for best health: a new national health research strategy: the NHS contribution to health research in England 2006 London: HMSO

[B4] Chalmers I Regulation of therapeutic research is compromising the interests of patients International Journal of Pharmaceutical Medicine 2007 21 395 404 10.2165/00124363-200721060-00004

[B5] Hawkes N The NHS stifles the entrepreneur in us all BMJ (Clinical research ed) 2007 335 913 10.1136/bmj.39381.648681.47 17974686 PMC2048864

[B6] Moynihan R Who pays for the pizza? Redefining the relationships between doctors and drug companies. 2: Disentanglement BMJ (Clinical research ed) 2003 326 1193 1196 10.1136/bmj.326.7400.1193 12775622 PMC1126054

[B7] Moynihan R Who pays for the pizza? Redefining the relationships between doctors and drug companies. 1: entanglement BMJ (Clinical research ed) 2003 326 1189 1192 10.1136/bmj.326.7400.1189 12775621 PMC1126053

[B8] Sheridan DJ Reforming research in the NHS BMJ (Clinical research ed) 2005 331 1339 1340 10.1136/bmj.331.7528.1339-c 16322034 PMC1298903

[B9] Department of Health Delivering Health Research: National Institute for Health Research - Progress Report 2008/09 London 2009

[B10] Medicines, Pharmacy and Industry Group Best practice guideline on joint working between the NHS and pharmaceutical industry and other relevant commercial organisations 2008 London: NHS, Department of Health

[B11] Cole A NHS research programme to be transformed BMJ (Clinical research ed) 2005 331 368 10.1136/bmj.331.7513.36816096300

[B12] Department of Health. The NHS Cancer Programme for England http://www.cancer.nhs.uk/index.htm

[B13] Centre for Medicines Research Global Clinical Performance Metrics Database http://www.cmr.org

[B14] Commercial Clinical Research in the UK A report for the Ministerial Industry Strategy Group (MISG) Clinical Research Working Group 2008 London: Kinapse Limited

[B15] NICE NHS Evidence - UK Database of Uncertainties about the Effects of Treatments (DUETs) 2009 http://www.library.nhs.uk/duets/

